# Genetic association study of selected candidate genes (ApoB, LPL, Leptin) and telomere length in obese and hypertensive individuals

**DOI:** 10.1186/1471-2350-10-99

**Published:** 2009-09-22

**Authors:** Birajalaxmi Das, Nilambari Pawar, Divyalakshmi Saini, M Seshadri

**Affiliations:** 1 Radiation Biology and Health Sciences Division, Bio-Medical Group, Bhabha Atomic Research Centre, Trombay, Mumbai 400 085, India; 2 School of Biotechnology, Chemical and Bio-medical Engineering, VIT University, Vellore, 632014, Tamilnadu, India

## Abstract

**Background:**

A genetic study was carried out among obese and hypertensive individuals from India to assess allelic association, if any, at three candidate loci: Apolipoprotein B (ApoB) minisatellite and two tetranucleotide repeat loci; LPL (Lipoprotein lipase) and Leptin. Attempt has also been made to find out whether telomere length attrition is associated with hypertension and obese individuals.

**Methods:**

Venous blood samples were collected from 37 normal, 35 obese and 47 hypertensive individuals. Genomic DNA was extracted from peripheral blood mononuclear cells (PBMC) and PCR amplifications were achieved using locus specific primers. Genotyping of ApoB minisatellite was performed using 4% polyacrylamide gel electrophoresis (PAGE) followed by silver staining, whereas LPL and Leptin loci were genotyped using ALF Express™ DNA sequencer. Telomere length was determined using a recently developed real time based quantitative PCR, where the relative telomere length was determined by calculating the relative ratio of telomere (T) and single copy gene (S) PCR products which is expressed as T/S ratio.

**Results:**

All the three loci are highly polymorphic, display high heterozygosity and conform to Hardy-Weinberg's equilibrium expectations. ApoB minisatellite displayed 14 alleles, whereas LPL and Leptin tetranucleotide loci were having 9 and 17 alleles, respectively. Interestingly two new alleles (9 and 11 repeats) were detected at ApoB locus for the first time. The alleles at Leptin locus were classified as Class I (lower alleles: 149-200 bp) and Class II alleles (higher alleles: >217 bp). Higher alleles at ApoB (>39 repeats), predominant allele 9 at LPL and alleles 164 bp and 224 bp at Leptin loci have shown allelic association with hypertensive individuals. After adjusting the influence of age and gender, the analysis of co-variance (ANCOVA) revealed the relative telomere length (T/S ratio) in hypertensive individuals to be (1.01 ± 0.021), which was significantly different (P < 0.001) from obese (1.20 ± 0.023) and normal (1.22 ± 0.014) individuals. However, no significant difference in the relative telomere length was observed among male and female individuals, although age related decrease in telomere length was observed in these limited sample size.

**Conclusion:**

The present study revealed that allelic association at ApoB, LPL, Leptin loci and loss of telomere length may have strong genetic association with hypertensive individuals. However, further study on larger sample size is needed to draw firm conclusions.

## Background

Essential hypertension and obesity both result from multiple environmental and genetic determinants. These disorders are known to be closely associated with high Body Mass Index (BMI) and have strong correlation with increased blood pressure. Interest in identifying the candidate genes or highly polymorphic tandemly repeated loci that contribute significantly to human obesity and essential hypertension is on the rise both in terms of designing of pharmacological intervention strategies and genetic association studies. Because there is a higher prevalence of both hypertension and obesity in modern human population, they represent excellent population for association studies. Tandemly repeated sequences of human genome such as minisatellites and microsatellites are highly variable and display a number of alleles in a population and thus considered as informative markers for association studies. ApoB minisatellite, LPL (Lipoprotein lipase) and Leptin tetranucleotide loci are good candidates for association studies as there are several reports showing that the alleles at these loci may be associated with hypertension, obesity and coronary heart diseases [1-7] The characteristic of ApoB minisatellite, LPL and Leptin tetranucleotides is given in table 1.

**Table 1:** Characteristics of the loci studied.

**Loci names**	**ApoB**	**LPL**	**LEPTIN**
Chromosome location	2p24	8p22	7q31.3
Repeat units (bps)	(TTTTATAATTAAATA)n	(TTTA)n	(TTTC)n
Product range (bps)	314-1050	105-145	148-288

bp = base pairs

Apolipoprotein B (ApoB) gene maps to 2p24 [8] and comprises 29 exons spanning about 42 kb [9]. Apolipoprotein B is the main apolipoprotein of chylomicrons and low density lipoproteins (LDL), which occurs in the plasma in 2 main forms, apoB48 and apoB100. ApoB-100 is synthesized in the liver and is present in very low density lipoproteins and their metabolic products. It is a principal ligand for low density lipoprotein (LDL) receptor [10]. LDL receptors mediate the uptake of LDL from the liver and peripheral cells; hence, Apo B-100 plays an important role in cholesterol homeostasis. A positive relationship between coronary heart disease and low density lipoprotein cholesterol with ApoB levels have been established [11]. The 3' end of the apo B gene exhibits a variable number of tandemly repeated (VNTR) short A+T rich DNA sequences [12]. Association of apoB 3' VNTR alleles and direct clinical diagnosis of essential hypertension was studied extensively [13]. Several alleles of this polymorphic locus have been found to be associated with coronary heart disease (CHD) and myocardial infarction as well as with various hyperlipidemias in different populations [14], thus can lead to severe obesity too.

LPL gene maps to chromosome 8p22 [15] and comprises 10 exons spanning about 30 kb [16,17]. Lipoprotein lipase (LPL), an enzyme plays a central role in the metabolism of lipoproteins by hydrolyzing the core triglycerides of circulating very low density lipoproteins (VLDL) and chylomicrons, thereby delivering lipoprotein derived fatty acids to adipose tissues for storage or oxidation in muscle [18,19]. Mutations in LPL or abnormal LPL lead to hypertriglyceridemia, dyslipidemia leading to various disorders like, coronary artery disease, hypertension, obesity etc. There are reports showing that abnormal adipose tissue LPL activity can lead to obesity in animal models and in humans [20,21]. Both Hypertriglyceridemia and dyslipidemia is a common finding in hypertensive patients and therefore LPL gene is considered as a logical candidate gene that could contribute to the development of hypertension [22,23]. LPL tetranucleotide locus spans intron 6 of LPL gene [24,25]. Significant evidence for linkage of systolic blood pressure, but not diastolic blood pressure has been associated with LPL locus located on the short arm of chromosome 8 (8p22) [26].

Leptin is called as human Obesity gene and it maps to 7q31.3 [27]. It consists of 3 exons and 2 introns, which spans approximately 18 kb. It is a 16-kD protein (hormone) mainly produced by adipose tissue that plays a critical role in the regulation of body weight by inhibiting food intake and stimulating energy expenditure [28]. Mutations in the gene encoding leptin are reported to cause severe obesity in animal models [29] and humans [30,31] indicating a direct relationship between leptin and obesity. In humans, mutations in the Leptin gene are a rare cause of obesity. But it has been a growing interest in determining whether polymorphic variation in or near the Leptin gene influences susceptibility to obesity in general population. The highly polymorphic Leptin tetranucleotide locus is located in 476 bp 3' of exon 3 of leptin gene [32] and has been associated with obesity [33] and hypertension [34]. Several researchers reported the evidence of linkage and/or association between variation in the Leptin gene region and traits related to obesity [35-39]. In the first part of our investigation we aimed at studying the genetic association if any, in hypertensive and obese individuals at three candidate loci as compared to Normal individuals.

Telomeres are DNA capping structures that protect the ends of eukaryotic chromosome. These are specialized nucleoprotein complexes and consist of tandem hexamer repeats of the sequence TTAGGG at the end of the chromosomes. They play an important role in maintaining chromosome stability as well as protecting of the coding parts of the DNA for recombination, degradation and replication damage. Telomere length is emerging as a biomarker for aging, stress and survival [40-42]. Several experimental methods have been developed to determine telomere length including southern blot, fluorescence in situ hybridization (Q-FISH) analysis [43], Flow-FISH analysis [44] and most recently, by quantitative PCR [45]. Quantitative PCR method provides relative results about telomere length by calculating the ratio of a PCR reaction product from the same sample using specific primers for telomeres and single copy gene (T/S ratio). Telomere attrition is reported to be associated with several diseases like diabetes [46,47], hypertension [48,49], coronary heart disease [50,51] and many cancers including bladder cancer [52]. Limited Studies are available on loss of telomere length and obesity [53]. Although shorter telomere length was found in Asian Diabetes patients [46] and Coronary Artery disease patients of Indian ethnicity [51], no Indian data is available till date on telomere length attrition in hypertensive and obese individuals.

In the present investigation, we have made an attempt to determine the telomere length in random, normal, healthy, adult individuals from hypertensive, obese and normal individuals and assess the telomere attrition if any, in hypertensive and obese individuals using real time quantitative PCR.

## Methods

### Sample collection

Approximately 5 ml of venous blood sample was collected in sterile EDTA-containing vacuutainers from a total of 121 random, individuals (69 males and 52 females) with informed consent which was approved by the ethic committee, Bhabha Atomic Research Centre, Trombay, Mumbai. These samples included 47 individuals with essential hypertension (systolic/diastolic BP range, 140/90 to 170/100; age range, 35 to 71 years), 35 individuals with obesity (Body Mass Index ≥ 30; age range, 11 to 60 years) and 39 normal individuals (systolic/diastolic BP range, 120/80 to 130/80 and Body Mass Index < 30; age range, 23 to 69) as controls. Blood pressure was measured using mercury sphygmomanometer.

The standard criteria adopted for hypertensive and obese individuals were as follows: Hypertension or high blood pressure is a medical condition, wherein the blood pressure is chronically elevated. The systolic/diastolic is equal to or under 120/80 is considered as normal individuals, whereas systolic/diastolic pressure >140/90 is considered as hypertensive. Another important criterion we adopted was all the hypertensive individuals studied here were not having diabetics or insulin resistance. Obesity is typically evaluated by measuring BMI (Body Mass Index), calculated by dividing the subject's weight in kilograms by the square of his/her height in meters (*BMI *= *kg*/*m*^2^). BMI of >30.0 is considered as obese. These obese individuals were also independent of diabetics and hypertension. All the individuals included in this study were nonsmokers.

### DNA extraction and PCR amplification

Genomic DNA was extracted from peripheral blood mononuclear cells (PBMC) using a rapid non-enzymatic method [54]. PCR amplification was achieved using locus specific primers as given in table 2. The forward primers were labeled with Cy5-dye amidite at the 5' end. The PCR mixture contained 20 pmols of each of the primers, 100 uM of dNTPs, 1× buffer (as per the manufacturer's protocol) and 0.5 units of taq polymerase enzyme (Roche diagnostics, GmbH, Germany). The PCR amplification protocol for ApoB is as follows: initial denaturation at 95°C for 5 minutes followed by a 30 cycles of melting 94°C for 1 minute, annealing at 55°C and extension at 72°C for one minute. For both LPL and Leptin loci, the PCR temperature conditions are as follows: initial denaturation at 95°C for 5 minutes followed by a 30 cycles of melting 94°C 30 seconds, annealing at 59°C for 30 sec and extension at 72°C for 30 sec with a final extension at 72°C for 5 minutes.

**Table 2:** Primer sequences.

**Locus name**	**Primer sequences**
ApoB PR1	5' ATGGAAACGGAGAAATTATG 3'
ApoB PR2	5' CCTTCTCACTTGGCAAATAC 3'
LPLPR1	5'CTGACCAAGGATAGTGGGATATAG3'
LPLPR2	5'GGTAACTGAGCGAGACTGTGTCT 3'
Leptin PR1	5'AGTTCAAATAGAGGTCCAAATCA3'
Leptin PR2	5' TTCTGAGGTTGTGTCACTGGCA 3'
Tel PR1	5'GGTTTTTGAGGGTGAGGGTGAGGGTGAGGGTGAGGGT 3'
Tel PR2	5' TCCCGACTATCCCTATCCCTATCCCTATCCCTATCCCTA 3'
36B4 PR1	5' CAGCAAGTGGGAAGGTGTAATCC 3'
36B4 PR2	5' CCCATTCTATCATCAACGGGTACAA **3'**
Beta globin PR1	5'GCTTCTGACACAACTGTGTTCACTAGC3'
Beta globin PR2	5'CACCAACTTCATCCACGTTCACC3'

PR1: Forward primer, PR2: Reverse primer

### Detection of alleles

**Figure 1:**
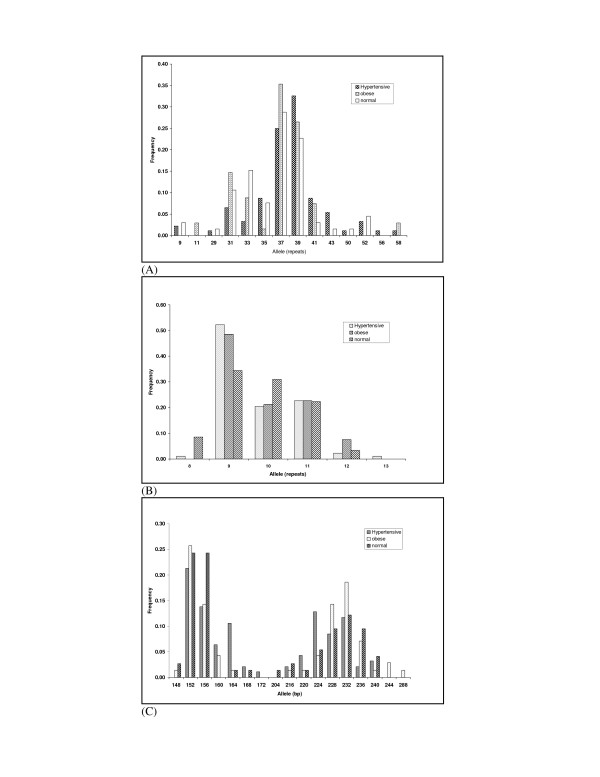
**Distribution of allele frequency in normal, obese and hypertensive individuals**. (A) ApoB ministaellite (B) LPL tetranucleotide locus (C) Leptin tetranucleotide locus.

The amplimers of ApoB locus were resolved in 4% native Poly Acrylamide Gel Electrophoresis (PAGE) followed by silver staining (figure 1A), whereas the alleles of LPL and Leptin loci were resolved in 6% denaturing PAGE using ALF Express™ DNA sequencer (GE Health Care, Uppsala, Sweden). The sizes of ApoB alleles were determined by using a software SEQAID [55], whereas LPL and Leptin alleles were determined using a software Fragment manager. The fluorogram showing LPL and Leptin alleles are given in figure 1B and 1C. External ladders 107 bp, 228 bp and 395 bp was used as external ladder. Internal ladders and allelic ladders were used for accurate size determination. Nomenclature of alleles refers to number of repeats i.e. allele 8 refers to 8 repeats.

### Relative telomere length analysis by real time quantitative PCR (q-PCR)

Telomere length was determined from a total of 93 individuals (58 males and 35 females) which included 26 normal, 27 obese and 40 hypertensive individuals. Relative telomere length was determined by using the approach as previously described by Cawthon in 2002 [45] with a little modification in the PCR temperature conditions. Relative telomere length was measured from genomic DNA obtained from peripheral blood mononuclear cells by quantitative real time polymerase chain reaction (PCR). This method measures the factor by which the ratio of telomere repeat copy number to single - gene copy number differs between a sample and that of a reference DNA sample. PCR amplification was achieved using telomere (T) and single copy gene, 36B4 (encodes acidic ribosomal phosphoprotein) primers (S) which serves as a quantitative control. The mean telomere repeat gene sequence (T) to a reference single copy gene (S) is represented as T/S ratio which is calculated to determine the relative telomere length. The expression of single copy gene (38B4) was validated using another positive control beta-globin gene. All the samples were run in triplicates in order to minimize the sample to sample variation. The error value indicates the degree of well to well variation in the 96 well plate used for the PCR experiment. The standard error between the replicate were approximately ≤0.05 (range: 0.02 to 0.05) in most of the samples. The telomere and single copy gene specific primers used for the experiment were as given in table 2.

Briefly, PCR reactions were performed in triplicate in 20 μl reaction volumes (using 25 ng DNA sample per reaction) for all the samples studied. The PCR reactions were performed using telomere and single copy gene primers in the same 96 well plate (LC480 light cycler from Roche diagnostics, GmbH, Germany). The PCR mixture contained 10 pmoles of each of the primers, 100 uM of each dNTPs and 0.3 × SYBR green dye and 0.5 Units of fast taq DNA polymerase (Roche Diagnostics, GmbH, Germany). The PCR thermal conditions for relative telomere length assay using telomeric primers (T) and single copy gene primers (S) consisted of a initial denaturation of 5 minutes at 95°C, followed by a total of 40 cycles at 95°C for 5 seconds, 56°C for 30 seconds, and 72°C for 30 seconds and fluorescence acquisition. Crossing points (Cp) were determined using the Light Cycler 480 software (Roche Diagnostics, GmbH, Germany). A standard curve derived from serially-diluted reference DNA was generated in order to check PCR efficiency between the plates. The average of telomere versus single copy gene (T/S) ratio was calculated which is proportional to telomere length of each individual as described by Cawthon et al 2002 [45]. For quality control purposes, we have repeated many samples that were separately PCR amplified. All measurements were performed in a blinded fashion without knowledge of the clinical data.

### Statistical analysis

The allele frequency, observed and expected heterozygosity, correspondence to Hardy-Weinberg's equilibria were calculated using software POPGENE32 (version 1.32) [56]. The expected heterozygosity was calculated under the assumption of Hardy-Weinberg equilibrium expectations [57]. Hardy-Weinberg equilibrium was evaluated using the likelihood ratio test (G-statistics). The power of discrimination (PD) was calculated as described by Fisher et al (1951) [58] and Polymorphic Information content (PIC) was calculated according to Botstein et al 1980 [59]. Association of alleles at each locus was determined by analyzing the significant increase of allele frequencies in hypertensive and obese individuals as compared to normal individuals.

Telomere length was determined after adjusting the influence of age and gender, if any, using analysis of Co-Variance (ANCOVA). Using analysis of variance (ANOVA), pairwise comparisons were performed in order to see the differences in the mean telomere length between the groups (normal, obese and hypertensive). Regression analysis was performed to find out the correlation between telomere length and age. ANOVA/ANCOVA, pair wise comparisons and regression analysis were performed using the software Statosoft [60].

## Results

In the present investigation, three highly polymorphic loci (one minisatellite, ApoB and two microsatellites; LPL and Leptin) were studied in normal, obese and hypertensive individuals. The distribution of the allele frequencies at these three loci is given in table 3 and represented as histograms in figures 1A, B, C. The statistical analysis for all the three loci is given in table 4.

**Table 3:** Distribution of allele frequencies at ApoB, LPL and Leptin loci in hypertensive, obese and normal individuals.

**Locus name**	**Allele**	**Hypertensive**	**Obese**	**Normal**
**ApoB**	(repeats)	Freq (obs. No)	Freq (obs. No)	Freq (obs. No)
	9	0.022 (2)		0.030 (2)
	11	-	0.029 (2)	-
	29	0.011 (1)	-	0.015 (1)
	31	0.065 (6)	0.147 (10)	0.106 (7)
	33	0.033 (3)	0.088 (6)	0.151 (10)
	35	0.087 (8)	0.015 (1)	0.076 (5)
	37	0.250 (23)	**0.353 **(24)	**0.288 **(19)
	39	**0.326 (30)**	0.265 (18)	0.227 (15)
	41	0.087 (8)	0.074 (5)	0.030 (2)
	43	**0.054 (5)**	-	0.015 (1)
	50	0.011 (1)	-	0.015 (1)
	52	0.033 (3)	-	0.046 (3)
	56	0.011 (1)	-	-
	58	0.011 (1)	0.029 (2)	-
		n = 92	n = 68	n = 66
				
**LPL**	(repeats)	Freq (obs. No.)	Freq (obs. No)	Freq (obs. No)
	8	0.011 (1)	-	0.086 (5)
	9	**0.523 (46)**	0.485 (32)	0.350 (20)
	10	0.205 (18)	0.212 (14)	0.310 (18)
	11	0.227 (20)	0.227 (15)	0.224 (13)
	12	0.023 (2)	0.076 (5)	0.035 (2)
	13	0.011 (1)	-	-
		n = 88	n = 66	n = 58
				
**Leptin**	(Base pairs)	Freq (obs. No.)	Freq (obs. No)	Freq (obs. No)
	148	-	0.014 (1)	0.027 (2)
	152	0.213 (20)	0.257 (18)	0.243 (18)
	156	0.138 (13)	0.143 (10)	0.243 (18)
	160	0.064 (6)	0.043 (3)	-
	164	**0.106 (10)**	0.014 (1)	0.014 (1)
	168	0.021 (2)	-	0.014 (1)
	172	0.011 (1)	-	-
	204	-	-	0.014 (1)
	216	0.021 (2)	0.041 (1)	0.027 (2)
	220	0.043 (4)	0.041 (1)	0.014 (1)
	224	**0.128 (12)**	0.043 (3)	0.054 (4)
	228	0.085 (8)	0.143 (10)	0.095 (7)
	232	0.117 (11)	0.186 (13)	0.122 (9)
	236	0.021 (2)	0.071 (5)	0.095 (7)
	240	0.032 (3)	0.041 (1)	0.041 (3)
	244	-	**0.029 (2)**	-
	288	-	**0.041 (1)**	-
		n = 94	n = 70	n = 74

n = Number of chromosomes, freq = Frequency, obs. No. = observed number

**Table 4:** Statistical Analysis of ApoB, LPL and Leptin loci in normotensive, obese and hypertensive individuals.

	**No. of alleles observed**	**Predominant Allele (repeat/bp)**	**No. of genotypes observed**	**Predominant genotype (repeat/bp)**	**Observed Heterozygosity**	**Expected Heterozygosity**	**PIC**	**PD**	**H-W equilibrium** **(P value)**
**ApoB locus**									
Normal (N = 33)	11	37	18	37-37	0.61	0.83	0.80	0.93	P = 0.623
Obese (N = 34)	8	37	15	37-37, 37-39, 39-39	0.62	0.78	0.74	0.91	P = 0.358
Hypertensive (N = 46)	13	39	24	37-37	0.63	0.83	0.78	0.92	P = 0.877
**LPL locus**									
Normal (N = 29)	5	9	9	9-9	0.83	0.74	0.68	0.84	P = 0.156
Obese (N = 33)	4	9	7	9-9	0.70	0.67	0.61	0.81	P = 0.094
Hypertensive (N = 44)	6	9	10	9-10, 9-11	0.62	0.64	0.58	0.81	P = 0.939
**Leptin locus**									
Normal (N = 29)	13	152, 156	23	152-156	0.76	0.85	0.82	0.94	P = 0.977
Obese (N = 33)	14	152	23	152-152	0.83	0.86	0.83	0.94	P = 0.999
Hypertensive (N = 44)	13	152	27	152-152, 224-228	0.77	0.88	0.87	0.94	P = 0.162

N = Number of samples, PIC = Polymorphic Information Content, PD = Power of Discrimination,

H-W:Hardy Weinberg

### ApoB locus

At ApoB locus a total of 14 alleles (11 in normal, 8 in obese and 13 in hypertensive) and 34 genotypes (tables 3, 4 and figure 1A) were observed. It showed a bimodal distribution with two predominant alleles (allele 37 and 39). Allele 39 was observed to be the most predominant allele among hypertensive individuals with a frequency of 0.326, while allele 37 was found to be the predominant one among obese and normal individuals with frequencies of 0.353 and 0.288, respectively (table 3). Allele 11 was exclusively found in obese individuals though at a lower frequency (0.029). Allele 58 was exclusively found in hypertensive and obese individuals, at a lower frequency of 0.011 and 0.029 respectively. Allele 56 was exclusively detected in hypertensive group at a frequency of 0.011. A total of 9 genotypes were exclusively present among hypertensive individuals and 3 genotypes were exclusively present among obese individuals as compared to normal individuals. The predominant genotype among hypertensive individuals was genotype 39-39, whereas genotype 37-37 was the predominant one among normal individuals. In contrast, there are three predominant genotypes (37-37, 37-39 and 39-39) among obese individuals (table 4).

The observed heterozygosity was found to be moderate (>0.61) in all the three groups. Under the assumption of Hardy Weinberg equilibrium expectations, the expected heterozygosity was calculated and was in the range of 0.78 to 0.83. The power of discriminations (PD) observed at this locus was >0.90. Polymorphism Information Content (PIC) of this locus was moderate and found to be in the range of 0.74-0.80 (table 4).

### LPL locus

At LPL locus, a total of six alleles (5 in normal, 4 in obese and 6 in hypertensive) and 12 genotypes were observed among these three groups (table 3, 4 and figure 1B). All the three groups showed trimodal distribution of allele 9, 10 and 11. However, allele 9 was found to be the most predominant allele among hypertensive, obese and normal individuals with frequencies of 0.523, 0.485 and 0.345, respectively. Allele 13 was exclusively observed among hypertensive individuals although at a lower frequency (0.011). A total of 10 genotypes were observed in hypertensive group, 7 in obese group and 9 in normal group (table 4). Predominant genotype was 9-9 in hypertensive and obese group whereas two predominant genotypes were found in normal individuals (9-10 and 9-11). There were two genotypes (8-10 and 11-13) observed exclusively observed among hypertensive individuals, although at a lower frequency.

At LPL locus, the observed heterozygosity had moderate values of 0.62 and 0.70 in hypertensive and obese groups, respectively and comparatively a high value of 0.83 for normal group. The expected heterozygosity was in the range of 0.64 to 0.74. Polymorphic Information Content (PIC) of this locus was moderate (range: 0.58 to 0.68), whereas the Power of Discrimination (PD) was observed to be >0.81 in all the three groups (table 4).

### Leptin locus

At Leptin locus a total of 17 alleles (13 in normal, 14 in Obese and 13 in hypertensive) and 49 different genotypes were observed (table 3, 4 and figure 1C). The most predominant allele in hypertensive and obese group was allele 152 bp with a frequency of 0.213 and 0.257, respectively, whereas the normal individuals showed a bimodal distribution at allele 152 bp and 156 bp with a similar frequency of 0.243. Allele 160 bp was exclusively observed among hypertensive and obese group though at a lower frequency. Alleles 244 bps and 288 bp were exclusively found among obese individuals. Allele 172 bp was exclusively found among hypertensive individuals (frequency of 0.011). The Predominant genotypes were found to 152-152 bp in obese (0.114) and 152-156 bp in normal (0.135) whereas hypertensive group showed two predominant genotypes (152-152 bp, 224-228) with a frequency of 0.106 each. Interestingly, there were 12 exclusive genotypes among hypertensive individuals and 10 exclusive genotypes among obese individuals (data not shown).

At this locus, all the alleles were grouped into two classes on the basis of its size (bp) distribution. Shorter alleles were named as class I alleles which ranges from 149 to 200 bp, whereas longer alleles were called as class II alleles, which were >217 bp as described by Shintani et al 1996 and 2002 [6,33]. On that basis, there are three genotypes namely, class I/I, II/II and I/II and the frequencies were given in table 5. The frequency of genotype I/I was significantly lower (P < 0.05) in obese individuals as compared to normal and hypertensive group. Genotypes II/II was observed at a marginally higher frequency among obese individual though not significant as compared to normal and hypertensive group. However, genotype I/II had a significantly higher frequency (P < 0.05) in obese group as compared to normal and hypertensive group.

**Table 5:** Distribution of genotype frequency at Leptin locus.

	**Hypertensive** **(N = 47)**	**Obese** **(N = 35)**	**Normotensive** **(N = 37)**
**Genotype (Class)**	**Observed Frequency**	**Observed Frequency**	**Observed Frequency**
I/I	0.383 (18)	0.257 (9)	0.351 (13)
II/II	0.277 (13)	0.314 (11)	0.270 (10)
I/II	0.340 (16)	0.429 (15)	0.378 (14)

The alleles of Leptin microsatellite polymorphism grouped into two classes with different size distribution: class I, 149-200 bp, class II, >217 bp. N = Number of individuals. Numbers in parantheses is the absolute number of observed frequency.

### Relative Telomere length

The relative telomere length was determined in a total of 93 individuals (58 males and 35 females). It included 26 normal, 27 obese and 40 hypertensive individuals. We have performed regression analysis to study the relationship between age and telomere length. Taking all the samples for consideration, our results showed a significant decrease in the mean telomere length (R = 0.5166991, P < 0.001) with respect to age. Similar trend was also observed when regression analysis of the telomere length with respect to age was performed among male and female individuals separately (male: R = 0.5216321, P < 0.001) and female (females: R = 0.5436911, P < 0.001). The relative telomere length was determined in male and female individuals from normal, obese and hypertensive groups. The mean telomere length was observed to be 1.15 ± 0.027 in males and 1.13 ± 0.029 in females, which were not statistically significant (P >0.05).

**Figure 2:**
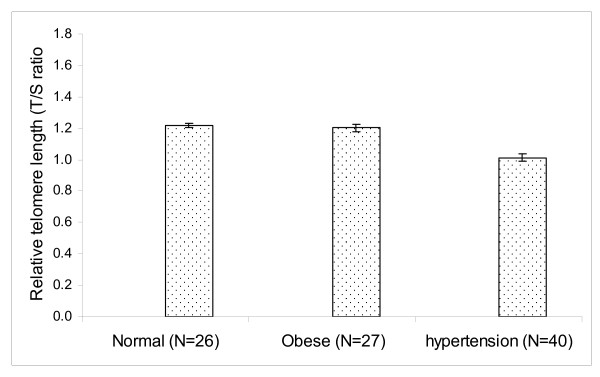
**Relative telomere length in normal, obese and hypertensive individuals**.

We have performed the analysis of covariance (ANCOVA) in order to adjust the influence of age and gender on telomere length. After adjusting the effect of age and gender, the mean telomere length was observed to be 1.22 ± 0.14 (95% CI, 1.19-1.25) in normal, 1.20 ± 0.023 (95% CI, 1.16-1.25) in obese and 1.01 ± 0.021 (95% CI, 0.97- 1.06) in hypertensive individuals (figure 2). The mean telomere length observed in hypertensive group was significantly different (P < 0.001) as compared to the values obtained from normal and obese groups. By taking gender alone as a variable the mean telomere length remained unchanged.

Pair-wise comparisons were performed taking all the three groups (normal, obese and hypertensive) into consideration in order to find out the difference between the groups, if any, with respect to telomere length. Our results revealed that hypertensive group was statistically different as compared to normal (p < 0.002) and obese (p < 0.001) groups.

## Discussion

Hypertension and obesity have strong genetic and multiple environmental determinants. The fact that the prevalence of hypertension and obesity is on the rise mostly due to life style, many genetic association studies have been undertaken all over the world by choosing suitable genetic markers from the candidate genes of hypertension and obesity. The multicentric study by Indian Council of Medical Research revealed that the prevalence rate in urban as well as rural areas in India is on the rise [61]. Considering the diversity and unique founder population in India, it is essential to carry out genetic association research on hypertension and obesity. In the present investigation, we report a genetic association study on obese and hypertension using three candidate loci (ApoB, LPL and Leptin). Since biological ageing has been associated with multifactorial diseases like essential hypertension and diabetes, we also have made an attempt to determine the leukocyte telomere length to find out the telomere length attrition, if any, in these individuals.

In the first part of our study we have compared the allele frequencies of ApoB, LPL and Leptin loci in hypertensive, obese groups as compared to normal individuals. This analysis has helped us to understand the allele specific association in the individuals studied. Interestingly, for the first time, we are reporting two new alleles (9 and 11 repeats) at ApoB locus. The predominant alleles (37 and 39 repeats) were found to be the same in normal, hypertensive and obese groups, which suggests that all the individuals belong to same founder population or with similar genetic background. Investigation at allele 35 at ApoB locus was significantly associated with clinical diagnosis of hypertensive patients in UAE Nationals [1] and reported to be advantageous in younger adults [2], whereas it could be dangerous in elders. DNA polymorphism study on various ethnic groups of Indian population [62] did not show allele 35 as the predominant allele at ApoB locus. Allele 35 did not seem to have any association in the present study population. We have observed approximately 21% alleles in hypertensive groups which were >39 repeats as compared to ~10% each in obese and normal individuals (p < 0.001). This suggests that higher alleles > 39 may have strong association with hypertension in this particular study group from India. There are reports which revealed that allele 50 (repeats) is associated with obese people [63]. But, we did not observe allelic association at allele 50 (repeats) with obese individuals. The large allelic variation and allele specific association to hypertension and obese individuals makes this informative locus suitable for genetic association studies.

At LPL locus, a trimodal distribution of alleles, (9, 10 and 11 repeats) in all the three groups showed the founder population/genetic similarity. The frequency of the most frequent allele (9 repeats) was significantly higher (p < 0.05) in hypertensive group as compared to normal individuals. In addition, allele 13 (repeats) was exclusively found among the hypertensive group. These finding at this locus is suggestive of allele specific association of this locus with hypertensive individuals. Earlier only five alleles were detected at this locus [25,64], but our study detected six alleles. The variability and allelic association of this informative locus also suggests that it will be useful for genetic association studies in diseases samples.

Leptin locus displayed high variability with large number of alleles (15 alleles) in our samples as compared to other populations in the world. Two classes of alleles: class I (shorter alleles) and Class II (longer alleles) was reported earlier [6,33]. Their study reported that the frequency of class I alleles was higher in hypertensive group as compared to control. The frequency of I/I genotype was significantly higher in hypertensive subjects independent of diabetes but not in obese [6]. It has also been reported that the frequency of class I alleles was significantly higher in obese as compared to normal individuals [5]. Especially the genotype I/II was significantly increased among obese as compared to normal and hypertensive individuals. In contrast, our study revealed a marginally lower frequency of class I/I genotype among obese as compared to normal and hypertensive individuals.

Another study had reported that leptin gene is not a major contributor of hypertension in African-Americans [7]. But in the present study, we report that genotype I/I occur at a similar frequency in normal and hypertensive individuals, which was significantly higher (p < 0.05) as compared to obese individuals. In obese individuals, the frequency of genotype II/II was significantly higher frequency (p < 0.001) as compared to normal and hypertensive individuals. This suggests that there may be a strong association of this genotype in obese individuals in Indian population. However, further investigation is required with larger number of samples using this polymorphic locus, which is a suitable candidate for genetic association studies in Indian population.

In the second part of our study, we have determined the telomere length among normal, obese and hypertensive individuals using a recently developed real time quantitative PCR. Since telomere length is an indicator of biological aging in humans [65,66], it is worth pursuing research on determining the causes of telomere attrition in age related diseases. Telomere attrition has been reported due to inflammation, exposure to infectious agents and other types of oxidative stress, which damage telomeres and impair their repair mechanisms [65,67]. It is also correlated with DNA damage response [68] and reported to be under genetic control which might play a role in mechanisms that regulate pulse pressure, including vascular aging [69].

Limited data is available on telomere shortening in hypertension and obese individuals. Shorter telomere length was observed in hypertensive individuals from Han Chinese [48] and Southern Taiwan [49] population. Similarly, loss of telomere length was reported in obese women from United States and Puerto Rico as compared to non obese-females [53]. In the present study, we report that hypertensive individuals have a significantly shorter telomere length (p < 0.001) as compared to normal and obese individuals. We did not observe shorter telomere length in obese individuals as compared to normal individuals. However, the causes of telomere shortening in hypertensive individuals are not known at this stage. Cumulative oxidative burden may affect telomere length in hypertensive individuals. So it is important to understand the biology of telomere attrition in multifactorial disease like hypertension.

Telomere length can also be affected by many confounding factors such as gender, lifestyle, diet, habits and stress. Associations between telomere length and stress/aging have significant implications for human health [41]. We observed a decreasing trend in telomere length with the increasing age. Since we have a limited sample size, the effect of various age groups on telomere length was not given separately for normal, obese and hypertensive individuals. Hence, we have analysed the telomere length in all the individuals after adjusting the age. There are studies showing significant difference in telomere length among male and female adults. A study has shown that the telomere length in females is significantly longer than males [42], although there are many studies which did not reveal any significant difference between the telomere length of male and female adults. In the present study, we did not find any significant loss of telomere length among male and female individuals in normal, obese and hypertensive groups. We have not included smokers in our study as smoking has been associated with telomere attrition [52,70].

### Perspectives

The present study illustrated association of some of the alleles at ApoB, LPL and Leptin loci with hypertensive and obese individuals in Indian population. The preliminary study with a limited sample has shown shorter telomere length in hypertensive individuals. Hence, further careful analysis on larger samples is required to throw some insight to the association of telomere length with hypertension and obese. Furthermore, this study will help in understanding the causative factor involved in leukocyte telomere biology. Telomere length regulation is essential for cell maintenance in humans, since telomere can lead to a number of defects including impaired cell division. Therefore studies on the mechanism of telomere length attrition in diseases like hypertension, obese and diabetes will provide immense understanding on its biological role.

## Conclusion

These three loci are excellent informative markers with immense potential to be used in genetic association studies in India. Our data would provide useful information to Indian population genetic databases as well as disease association studies related to obese and hypertensive individuals. Telomere length measurement using real time PCR did not reveal any significant loss of telomere length in obese individuals, whereas, there may be association between the telomere length and hypertensive individuals. However, sample size should be increased to give a better understanding of the disease association and population genetic studies. To our knowledge, this study is the first report from Indian population dealing with association of alleles with hypertension and obese and telomere length determination in hypertensive and obese individuals using quantitative real time PCR.

## Competing interests

The authors declare that they have no competing interests.

## Authors' contributions

The project is conceived and implemented by BD. The genotyping of the loci was done by NP, Real time PCR experiment for telomere length was done by BD and DS. Data analysis, interpretation and writing of the manuscript were by BD. Overall supervision and discussion was by MS. All the authors read and approved the final manuscript.

## Pre-publication history

The pre-publication history for this paper can be accessed here:



## References

[B1] Frossard PM, Obineche EN, Lestringant GG (1999). Analysis of the Apolipoprotein B gene 3' hypervariable region among nationals of the Abu Dhabi emirate and comparisons with other populations. Annals of Saudi Medicine.

[B2] Garasto S, Berrardelli M, DeRango F, Mari V, Feraco E, De Benedictis G (2004). A study of the average effect of the 3' APOB-VNTR polymorphism on lipidemic parameters could explain why the short alleles (<35 repeats) are rare in centenarians. BMC Medical Genetics.

[B3] Ruixing Y, Guangqin C, Yong W, Weixiong L, Dezhai Y, Shangling P (2007). Effect of the 3' APOB-VNTR polymorphism on the lipid profiles in the Guangxi Hei Yi Zhuang and Han populations. BMC Medical Genetics.

[B4] Maruyama S, Minaguchi K (2005). Polymorphism of LPL locus in Japanese and comparison of PCR amplification efficiency from degraded DNA between LPL locus and D21S11. Bull Tokyo Dent Coll.

[B5] McGarvey ST, Forrest W, Weeks DE, Sun G, Smelser D, Tufa J, Viali S, Deka R (2002). Human Leptin locus (LEP) alleles and BMI Samoans. International Journal of Obesity.

[B6] Shintani M, Hiroshi I, Tomomi F, Yoshihiko K, Mitsuru O, Tomohiro K, Jitsuo H, Kazuaki S, Toshio O (2002). Leptin Gene Polymorphism Is Associated with Hypertension Independent of Obesity. The Journal of Clinical Endocrinology & Metabolism.

[B7] Onions KL, Hunt SC, Rutkowski MP, Klanke CA, Su YR, Reif M, Menon AG (2007). Genetic markers at Leptin (OB) locus are not significantly linked to hypertension in African Americans. Hypertension.

[B8] Knott TJ, Rall SCJr, Innerarity TL, Jacobson SF, Urdea MS, Levy-Wilson B, Powell LM, Pease RJ, Eddy R, Nakai H, Byers M, Priestley LM, Robertson E, Rall LB, Betsholtz C, Shows TB, Mahley RW, Scott J (1985). Human apolipoprotein B: structure of carboxyl-terminal domains, sites of gene expression, and chromosomal localization. Science.

[B9] Blackhart BD, Ludwig EM, Pierotti VR, Caiati L, Onasch MA, Wallis SC, Powell L, Pease R, Knott TJ, Chu ML (1986). Structure of the human apolipoprotein B gene. Biol Chem.

[B10] Boerwinkle E, Xiong W, Fourest E, Chan L (1989). Rapid typing if tandemly repeated hypervariable loci by the polymerase chain reaction: application to the apolipoprotein B 3' hypervariable region. Proc Natl Acad Sci USA.

[B11] Brunzell JD, Sniderman AD, Albers JJ, Kwiterovich PO (1984). Apoproteins B and AI and coronary artery disease in humans. Arteriosclerosis.

[B12] Knott TJ, Wallis SC, Powell LM, Pease RJ, Lusis AJ, Blackhart B, McCarthy BJ, Wilson B, Scott J (1986). A hypervariable region 3' to the human apolipoprotein B gene. Nucleic acids research.

[B13] Philippe MF, Enyioma N, Obineche G, Lestringant G (1999). Association of an Apolipoprotein B Gene Marker with Essential Hypertension. Hypertension.

[B14] Friedl W, Ludwig EH, Paulweber B, Sandhofer F, McCarthy BJ (1990). Hypervariability in a minisatellite 3' of the apolipoprotein B gene in patients with coronary heart disease compared with normal controls. J Lipid Res.

[B15] Sparkes RS, Zollman S, Klisak I, Kirchgessner TG, Komaromy MC, Mohandas T, Schotz MC, Lusis AJ (1987). Human genes involved in lipolysis of plasma lipoproteins: mapping of loci for lipoprotein lipase to 8p22 and hepatic lipase to 15q21. Genomics.

[B16] Deeb SS, Peng R (1989). Structure of the human lipoprotein lipase gene. Biochemistry.

[B17] Monsalve MV, Henderson H, Roederer G, Julien P, Deeb S, Kastelein JJP, Peritz L, Devlin R, Bruin T, Murthy MRV, Gagne C, Davignon J, Lupien PJ, Brunzell JD, Hayden MR (1990). A missense mutation at codon 188 of the human lipoprotein lipase gene is a frequent cause of lipoprotein lipase deficiency in persons of different ancestries. J Clin Invest.

[B18] Jackson RL (1983). Lipoprotein lipase and hepatic lipase. The Enzymes.

[B19] Garfinkel AS, Schotz MC, Gotto AM (1987). Lipoprotein lipase. In Plasma Lipoproteins.

[B20] Gruen R, Hietanen E, Greenwood M, Greenwood R (1978). Increased adipose tissue lipoprotein lipase activity during the development of the genetically obese rat *(fa/fa)*. Metabolism.

[B21] Lithell H, Boberg J (1978). The lipoprotein-lipase activity of adipose tissue from different sites in obese women and relationship to cell size". International Journal of Obesity.

[B22] Chen P, Jou YS, Fann CS, Chen JW, Wu SY, Pan WH (2005). Lipoprotein lipase gene is linked and associated with hypertension in Taiwan young-onset hypertension genetic study. J Biomed Sci.

[B23] Yang W, Huang J, Ge D, Yao C, Duan X, Gan W, Huang G, Zhao J, Hui R, Shen Y, Qiang B, Gu D (2003). Variation near the region of the lipoprotein lipase gene and hypertension or blood pressure levels in Chinese. Hypertens Res.

[B24] Oka K, Tkalcevic GT, Stocks J, Galton DJ, Brown WV (1989). Nucleotide sequence of PvuII polymorphic site at the lipoprotein lipase gene locus. Nucleic Acids Res.

[B25] Zuliani G, Hobbs HH (1990). Tetranucleotide repeat polymorphism in the LPL gene. Nucleic Acids Res.

[B26] Du-An W, Xiangdong B, Craig H, Warden D, Shen DC, Jeng CY, Wayne HH, Sheu M, Fuh MT, Tomohiro K, Victor JD, Gerald MR, Aldons JL, Jerome IR, Chen YD (1992). Quantitative Trait Locus Mapping of Human Blood Pressure to a Genetic Region at or near the Lipoprotein Lipase Gene Locus on Chromosome 8p22. J Clin Invest.

[B27] Isse N, Ogawa Y, Tamura N, Masuzaki H, Mori K, Okazaki T, Satoh N, Shigemoto M, Yoshimasa Y, Nishi S, Hosoda K, Inazawa J, Nakao K (1995). Structural organization and chromosomal assignment of the human obese gene. J Biol Chem.

[B28] Campfield LA, Smith FJ, Burn P (1996). The Ob protein (leptin) pathway: a link between adipose tissue mass and central neural networks. Horm Metab Res.

[B29] Zhang Y, Proenca R, Maffei M, Barone M, Leopold L, Friedman JM (1994). Positional cloning of the mouse obese gene and its human homologue. Nature.

[B30] Montague CT, Farooqi IS, Whitehead JP, Soos MA, Rau H, Wareham NJ, Sewter CP, Digby JE, Mohammed SN, Hurst JA, Cheetham CH, Earley AR, Barnett AH, Prins JB, O'Rahilly S (1997). Congenital leptin deficiency is associated with severe early-onset obesity in humans. Nature.

[B31] Strobel A, Issad T, Camoin L, Ozata M, Strosberg AD (1998). A leptin missense mutation associated with hypogonadism and morbid obesity. Nat Genet.

[B32] Moffett S, Martinson J, Mark DS, Deka R, McGarvey ST, Barrantes R, Ferrell RE (2002). Genetic diversity and evolution of the human leptin locus tetranucleotide repeat. Hum Genet.

[B33] Shintani M, Ikegami H, Yamato E, Kawaguchi Y, Fujisawa T, Nakagawa Y, Hamada Y, Ueda H, Miki T, Ogihara T (1996). A novel microsatellite polymorphism in the human OB gene: a highly polymorphic marker for linkage analysis. Diabetologia.

[B34] Borecki IB, Rice T, Perusse L, Bouchard C, Rao DC (1994). An exploratory investigation of genetic linkage with body composition and fatness phenotypes: the Quebec Family study. Obes Res.

[B35] Clement K, Garner C, Hager J, Philippi A, LeDuc C, Carey A, Harris TJ, Jury C, Cardon LR, Basdevant A, North M, Froguel P (1996). Indication for linkage of the human OB gene region with extreme obesity. Diabetes.

[B36] Duggirala R, Stern MP, Mitchell BD, Reinhart LJ, Shipman PA, Uresandi OC, Chung WK, Hales CN, Blangero J (1996). Quantitative variation in obesity related traits and insulin precursors linked to the OB gene region on human chromosome 7. American journal of Hum Genet.

[B37] Reed DR, Ding Y, Xu W, Cather C, Green ED, Price RA (1994). Extreme obesity may be linked to markers flanking the human OB gene. Diabetes.

[B38] Bray MS, Boerwinkle E, Hanis CL (1996). OB gene not linked to human obesity in Mexican American affected sib pairs from Starr County. Hum Genet.

[B39] Hasstedt SJ, Hoffman M, Leppert MF, Elbein SC (1997). Recessive inheritance of obesity in familial non-insulin dependent diabetes mellitus, and lack of linkage to nine candidate genes. Am J Hum Genet.

[B40] Njajou OT, Cawthon RM, Damcott CM, Shih-Hsuan W, Ott S, Garant MJ, Blackburn EH, Mitchell BD, Shuldiner AR, Hsueh WC (2007). Telomere length is paternally inherited and is associated with parental lifespan. PNAS.

[B41] Epel ES, Blackburn EH, Lin J, Dhabhar FS, Adler NE, Morrow JD, Cawthon RM (2004). Accelerated telomere shortening in response to life stress. Proceedings of National Academy of Sciences.

[B42] Benetos A, Okuda K, Lajemi M, Kimura M, Thomas F, Skurnick J, Labat C, Bean K, Aviv A (2001). Telomere length as an indicator of biological aging: The gender effect and relation with pulse pressure and pulse wave velocity. Hypertension.

[B43] Poon SS, MaartensSigma UM, Ward RK, Lansdrop PM (1999). Telomere length measurements using digital fluorescence microscopy. Cytometry.

[B44] Rufer N, Dragwoska W, Thornbery G, Roosneck E, Lansdorp PM (1998). Telomere length dynamics in human lymphocyte subpopulations measured by flow cytometry. Nature Biotech.

[B45] Cawthon RM (2002). Telomere measurement by quantitative PCR. Nucleic Acids Res.

[B46] Adaikalakoteswari A, Balasubramanyam M, Mohan V (2005). Telomere shortening occurs in Asian Indian Type 2 diabetic patients. Diabetic medicine.

[B47] Sampson MJ, Winterbone MS, Hughes MS, Dozio JC, Nicoletta D, Hughes DA (2006). Monocyte Telomere Shortening and Oxidative DNA Damage in Type 2 Diabetes. Diabetes Care.

[B48] Lung FW, Ku CS, Kao WT (2008). Telomere length may be associated with hypertension. Journal of human hypertension.

[B49] Yang Z, Huang X, Jiang H, Zhang Y, Liu H, Qin C, Eisner GM, Jose P, Rudolph L, Ju Z (2009). Short Telomeres and Prognosis of Hypertension in a Chinese Population. Hypertension.

[B50] Fitzpatrick AL, Kronmal RA, Gardner JP, Psaty BM, Jenny NS, Tracy RP, Walston J, Kimura M, Aviv A (2006). Leukocyte Telomere Length and Cardiovascular Disease in the Cardiovascular Health Study. Am J Epidemiol.

[B51] Mukherjee M, Brouilette S, Stevens S, Shetty KR, Samani NJ (2009). Association of shorter telomeres with coronary artery disease in Indian subjects. Heart.

[B52] McGrath M, Wong JYY, Michaud D, Hunter DJ, De vivo I (2007). Telomere length, cigarette smoking and bladder cancer risk in men and women. Cancer epidemiology biomarkers prevention.

[B53] Kim S, Parks CG, DeRoo LA, Chen H, Taylor JA, Cawthon RM nd Sandler DP (2009). Obesity and Weight Gain in Adulthood and Telomere Length. Cancer Epidemiol Biomarkers Prev.

[B54] Lahiri K, Nurnberger JI (1991). A rapid non-enzymatic method for the preparation of HMW DNA from blood for RFLP studies. Nucleic acids Research.

[B55] Peltoal H, Soderlund H, Ukkonen E (1984). SEQAID: a DNA sequence assembling program based on a mathematical model. Nucleic Acids Res.

[B56] Yeh FC, Yang RC, Boyle T (1999). POPGENE (version 1.32). http://www.ualberta.ca/~fyeh.

[B57] Nei M (1978). Estimation of average heterozygosity and genetic distance from a small number of individuals. Genetics.

[B58] Fisher R (1951). Standard calculations for evaluating a blood group system. Heredity.

[B59] Botstein D, White RL, Skolnick M, Davis RW (1980). Construction of a genetic linkage map in man using in man using restriction length polymorphism. American journal of Human genetics.

[B60] Statosoft Inc (1995). STATISTICA for Windows. Tulsa, OK, USA.

[B61] Hypertension study Group (2001). Prevalence, Awareness, treatment and control of hypertension among elderly in Bangladesh and India: a multicentric study. Bulletin of the World Health Organization.

[B62] Das B, Ghosh A, Chauhan PS, Seshadri M (2002). Genetic polymorphism study at four minisatellite loci (D1S80, D17S5, D19S20 and APOB) among five Indian population groups. Human Biology.

[B63] Jemaa R, El-Asmi M, Mebazaa A (2002). VNTR3' polymorphism of apolipoprotein B gene in obese people. Ann Biol clin.

[B64] Young IA, Kamboh MI, Robert EF (1992). Two new alleles in the tetranucleotide repeat polymorphism at the lipoprotein lipase locus. Hum Genet.

[B65] Aviv A (2002). Chronology Versus Biology: Telomeres, Essential Hypertension, and Vascular ageing. Hypertension.

[B66] Ilmonen P, Kotrschal A, Penn SJ (2008). Telomere attrition due to infection. PLoS One.

[B67] Aviv A, Valdes AM, Spector TD (2006). Human telomere Biology: Pitfalls of moving from the laboratory to epidemiology. Int J Epidemiology.

[B68] Raynaud CM, Jang SJ, Nuciforo P, Lantuejoul S, Brambilla E, Mounier N, Olaussen KA, André F, Morat L, Sabatier L, Soria JC (2008). Telomere shortening is correlated with the DNA damage response and telomeric protein down-regulation in colorectal preneoplastic lesions. Annals of Oncology.

[B69] Jeanclos E, Schork NJ, Kyvik KO, Kimura M, Skurnick JH, Aviv A (2000). Telomere Length Inversely Correlates With Pulse Pressure and Is Highly Familial. Hypertension.

[B70] Valdes AM, Andrew T, Gardner JP, Kimura M, Oelsner E, Cherkas LF, Aviv A, Spector TD (2005). Obesity, cigarette smoking, and telomere length in women. The Lancet.

